# Supplementary material for: Evolutionary selection of proteins with two folds
Source: Nat Commun. 2023 Sep 6;14:5478. doi: 10.1038/s41467-023-41237-2 (PMC10482954; doi:10.1038/s41467-023-41237-2)
Supplement: Supplementary file 1 — Supplementary Information [file 41467_2023_41237_MOESM1_ESM.pdf]

Supplementary Information for

Evolutionary selection of proteins with two folds

[1]Joseph W. Schafer and [1,2]Lauren L. Porter*

[1]National Library of Medicine, National Center for Biotechnology Information, National Institutes of Health, Bethesda, MD 20894, USA

[2]National Heart, Lung, and Blood Institute, Biochemistry and Biophysics Center, National Institutes of Health, Bethesda, MD 20892, USA

*Corresponding Author: lauren.porter@nih.gov

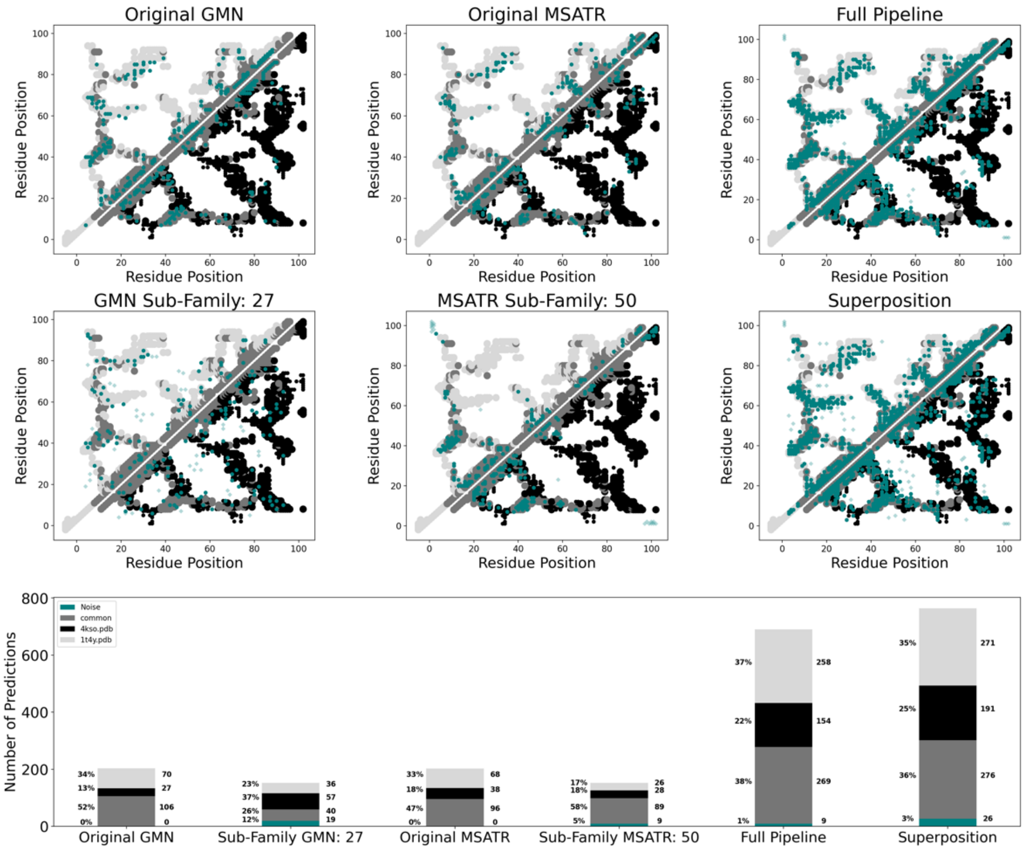

Supplementary Figure 1: Our approach enhances predictions of coevolved amino acid pairs: example 1 of 10. All six upper panels show predicted contacts referenced against contacts from two experimentally determined structures of KaiB with PDB codes 5JYT (dominant conformation) and 4KSO (alternative conformation). Color schemes of these six panels follow: experimentally determined contacts unique to the dominant conformation (light gray), alternative conformation (black), common to both conformations (dark gray), interchain contacts from homomers (small circles), predicted contacts (teal) corresponding to experimentally determined structures (opaque circles), predicted contacts not corresponding to experimentally determined structures (noise, translucent diamonds). Panels, top-to-bottom, left-to-right show the experimentally determined contacts underlaying: GREMLIN predictions from the original MSA (Original GMN) and the shallowest subfamily MSA (GMN subfamily, 25 indicates minimum %sequence identity to query); MSA Transformer predictions from the original MSA (Original MSATR) and the shallowest subfamily MSA (MSATR subfamily, 49 indicates minimum %sequence identity to query); predictions from our approach after noise filtering (Full Pipeline) and before noise filtering (Superposition). Predicted contacts from each panel are tabulated by overlap with experimentally determined contacts in the bar graph: dominant (light gray); alternative (black); common (dark gray); noise (teal). Predicted contacts from GREMLIN and MSA Transformer are often redundant; signal increases from our approach are shown in Supplementary Figure 12.

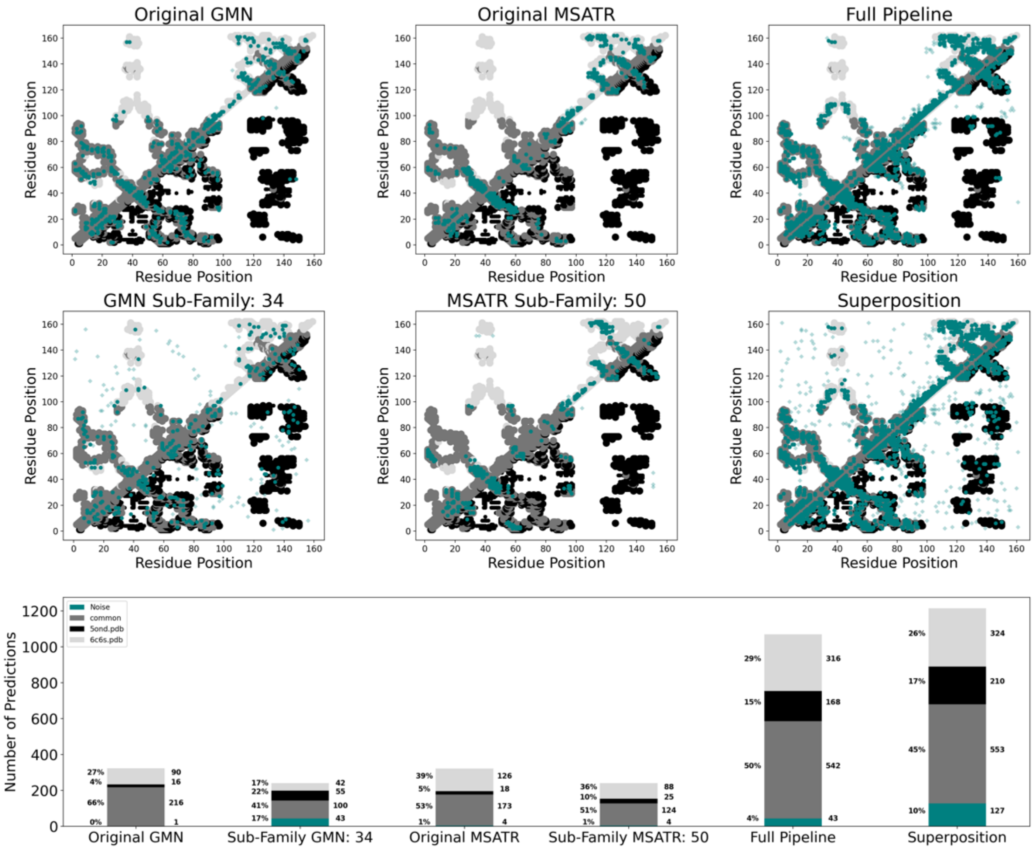

Supplementary Figure 2: Our approach enhances predictions of coevolved amino acid pairs: example 2 of 10. All six upper panels show predicted contacts referenced against contacts from two experimentally determined structures of RfaH with PDB codes 6C6S (dominant conformation) and 5OND (alternative conformation). Color schemes of these six panels follow: experimentally determined contacts unique to the dominant conformation (light gray), alternative conformation (black), common to both conformations (dark gray), interchain contacts from homomers (small circles); predicted contacts (teal) corresponding to experimentally determined structures (opaque circles), predicted contacts not corresponding to experimentally determined structures (noise, translucent diamonds). Panels, top-to-bottom, left-to-right show the experimentally determined contacts underlaying: GREMLIN predictions from the original MSA (Original GMN) and the shallowest subfamily MSA (GMN subfamily, 33 indicates minimum %sequence identity to query); MSA Transformer predictions from the original MSA (Original MSATR) and the shallowest subfamily MSA (MSATR subfamily, 38 indicates minimum %sequence identity to query); predictions from our approach after noise filtering (Full Pipeline) and before noise filtering (Superposition). Predicted contacts from each panel are tabulated by overlap with experimentally determined contacts in the bar graph: dominant (light gray); alternative (black); common (dark gray); noise (teal). Predicted contacts from GREMLIN and MSA Transformer are often redundant; signal increases from our approach are shown in Supplementary Figure 12

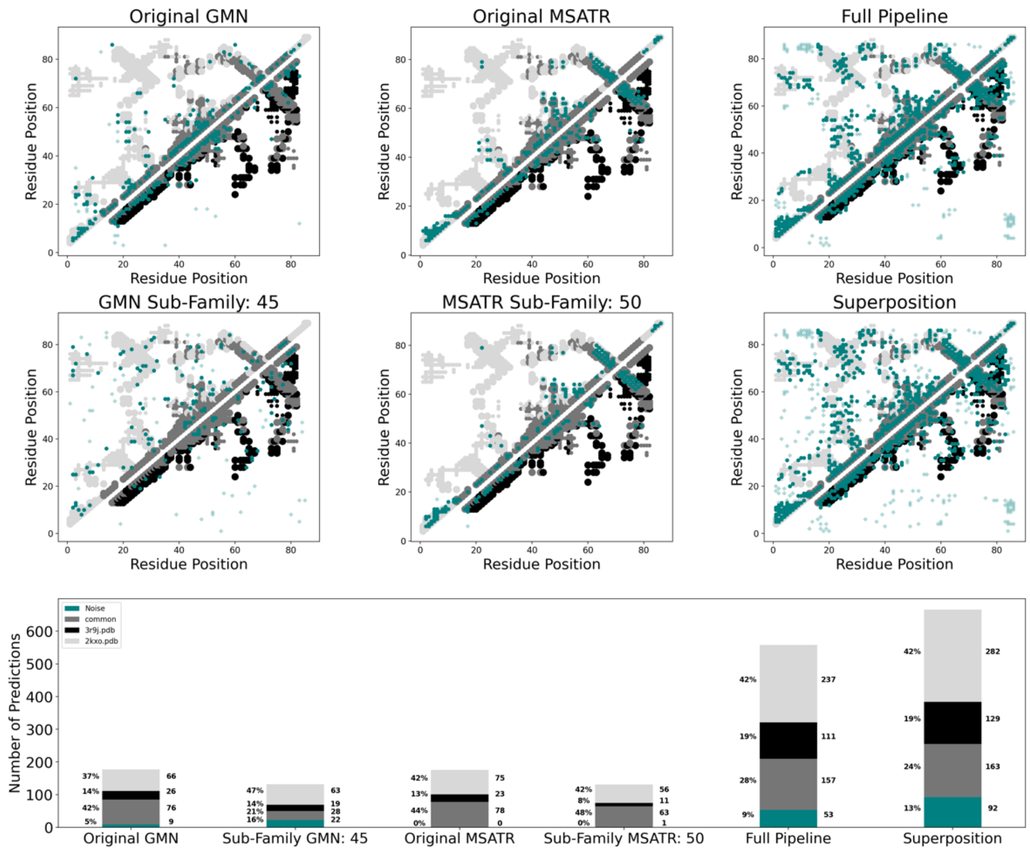

Supplementary Figure 3: Our approach enhances predictions of coevolved amino acid pairs: example 3 of 10. All six upper panels show predicted contacts referenced against contacts from two experimentally determined structures of MinE with PDB codes 2KXO (dominant conformation) and 3R9J (alternative conformation). Color schemes of these six panels follow: experimentally determined contacts unique to the dominant conformation (light gray), alternative conformation (black), common to both conformations (dark gray), interchain contacts from homomers (small circles); predicted contacts (teal) corresponding to experimentally determined structures (opaque circles), predicted contacts not corresponding to experimentally determined structures (noise, translucent diamonds). Panels, top-to-bottom, left-to-right show the experimentally determined contacts underlaying: GREMLIN predictions from the original MSA (Original GMN) and the shallowest subfamily MSA (GMN subfamily, 43 indicates minimum %sequence identity to query); MSA Transformer predictions from the original MSA (Original MSATR) and the shallowest subfamily MSA (MSATR subfamily, 47 indicates minimum %sequence identity to query); predictions from our approach after noise filtering (Full Pipeline) and before noise filtering (Superposition). Predicted contacts from each panel are tabulated by overlap with experimentally determined contacts in the bar graph: dominant (light gray); alternative (black); common (dark gray); noise (teal). Predicted contacts from GREMLIN and MSA Transformer are often redundant; signal increases from our approach are shown in Supplementary Figure 12.

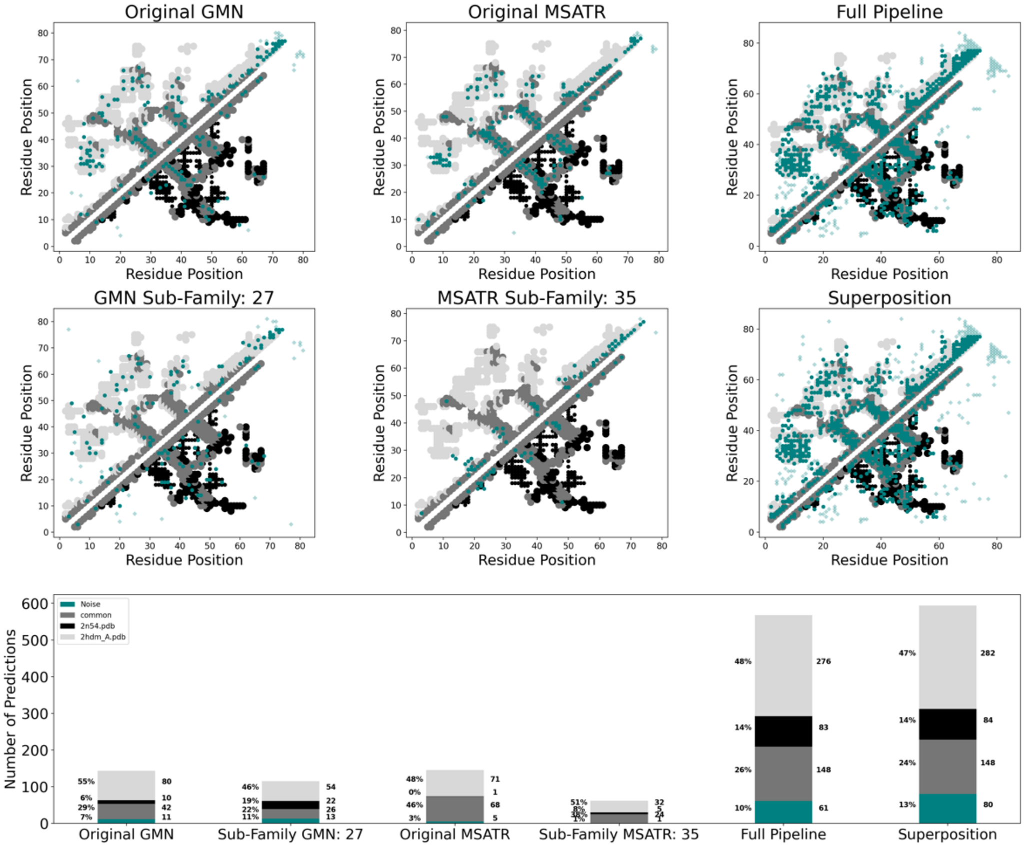

Supplementary Figure 4: Our approach enhances predictions of coevolved amino acid pairs: example 4 of 10. All six upper panels show predicted contacts referenced against contacts from two experimentally determined structures of XCL1 with PDB codes 2HDM (dominant conformation) and 2N54 (alternative conformation). Color schemes of these six panels follow: experimentally determined contacts unique to the dominant conformation (light gray), alternative conformation (black), common to both conformations (dark gray), interchain contacts from homomers (small circles); predicted contacts (teal) corresponding to experimentally determined structures (opaque circles), predicted contacts not corresponding to experimentally determined structures (noise, translucent diamonds). Panels, top-to-bottom, left-to-right show the experimentally determined contacts underlaying: GREMLIN predictions from the original MSA (Original GMN) and the shallowest subfamily MSA (GMN subfamily, 28 indicates minimum %sequence identity to query); MSA Transformer predictions from the original MSA (Original MSATR) and the shallowest subfamily MSA (MSATR subfamily, 38 indicates minimum %sequence identity to query); predictions from our approach after noise filtering (Full Pipeline) and before noise filtering (Superposition). Predicted contacts from each panel are tabulated by overlap with experimentally determined contacts in the bar graph: dominant (light gray); alternative (black); common (dark gray); noise (teal). Predicted contacts from GREMLIN and MSA Transformer are often redundant; signal increases from our approach are shown in Supplementary Figure 12.

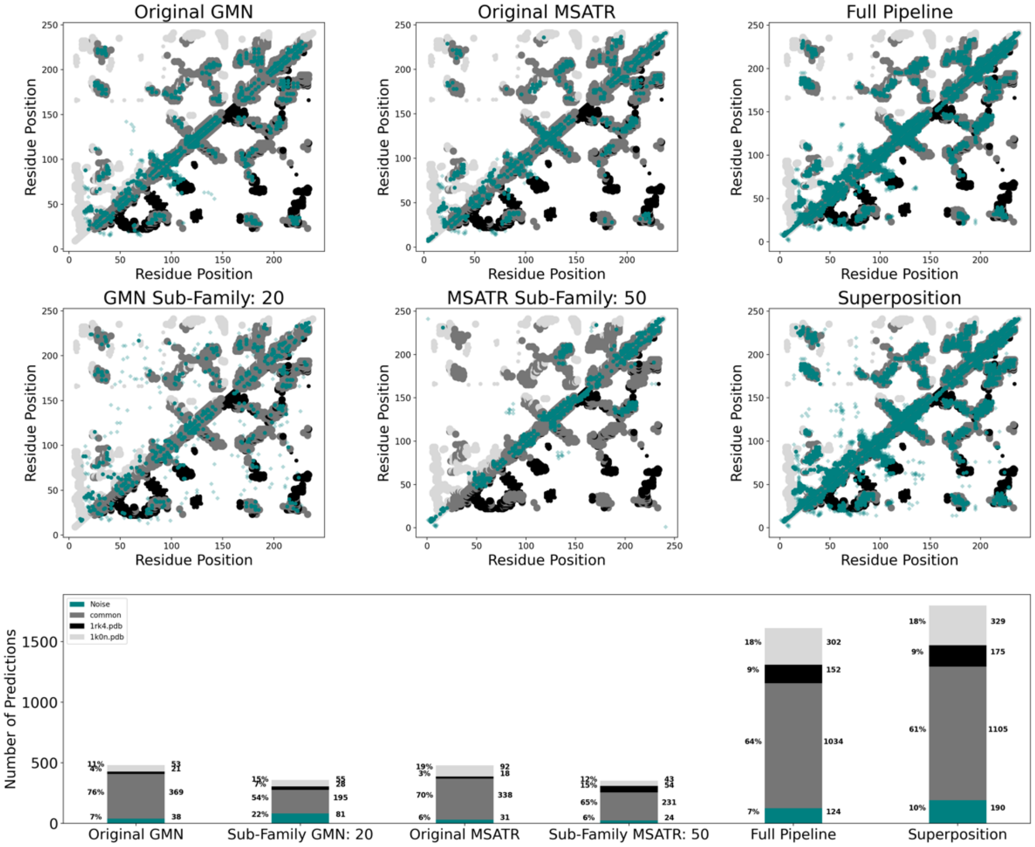

Supplementary Figure 5: Our approach enhances predictions of coevolved amino acid pairs: example 5 of 10. All six upper panels show predicted contacts referenced against contacts from two experimentally determined structures of CLIC1 with PDB codes 1K0N (dominant conformation) and 1RK4 (alternative conformation). Color schemes of these six panels follow: experimentally determined contacts unique to the dominant conformation (light gray), alternative conformation (black), common to both conformations (dark gray), interchain contacts from homomers (small circles); predicted contacts (teal) corresponding to experimentally determined structures (opaque circles), predicted contacts not corresponding to experimentally determined structures (noise, translucent diamonds). Panels, top-to-bottom, left-to-right show the experimentally determined contacts underlaying: GREMLIN predictions from the original MSA (Original GMN) and the shallowest subfamily MSA (GMN subfamily, 21 indicates minimum %sequence identity to query); MSA Transformer predictions from the original MSA (Original MSATR) and the shallowest subfamily MSA (MSATR subfamily, 23 indicates minimum %sequence identity to query); predictions from our approach after noise filtering (Full Pipeline) and before noise filtering (Superposition). Predicted contacts from each panel are tabulated by overlap with experimentally determined contacts in the bar graph: dominant (light gray); alternative (black); common (dark gray); noise (teal). Predicted contacts from GREMLIN and MSA Transformer are often redundant; signal increases from our approach are shown in Supplementary Figure 12.

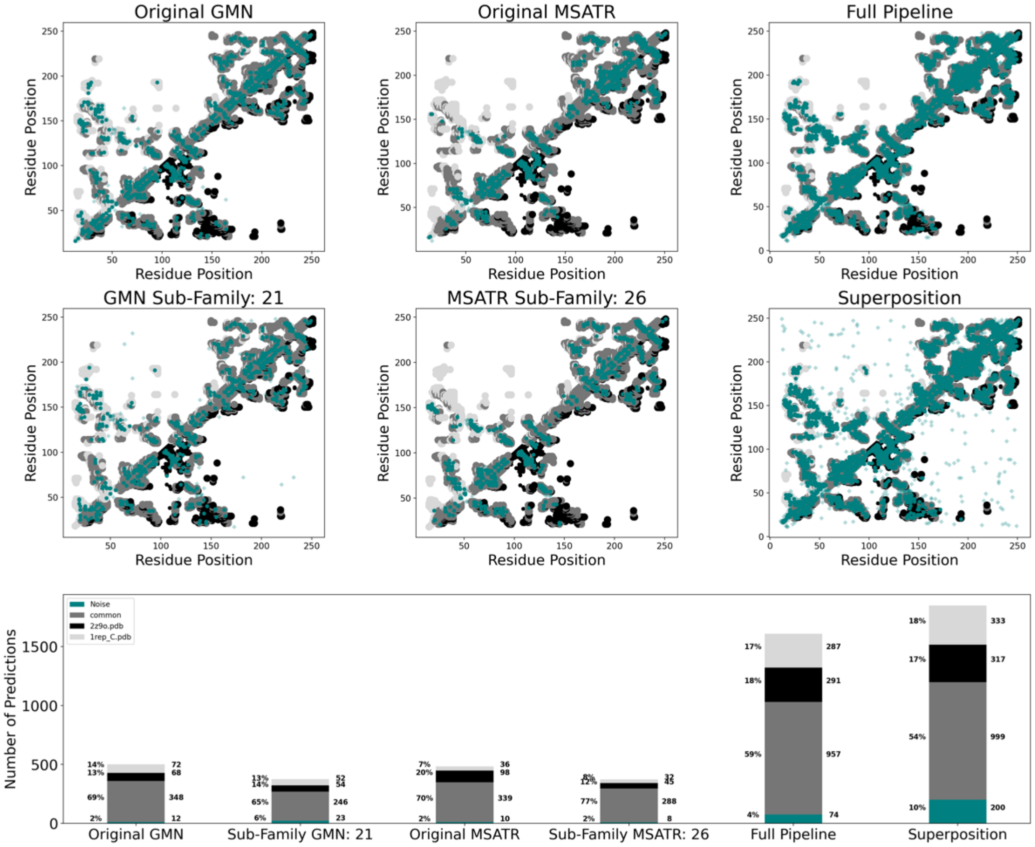

Supplementary Figure 6: Our approach enhances predictions of coevolved amino acid pairs: example 6 of 10. All six upper panels show predicted contacts referenced against contacts from two experimentally determined structures of RepE with PDB codes 2Z9O (dominant conformation) and 1REP (alternative conformation). Color schemes of these six panels follow: experimentally determined contacts unique to the dominant conformation (light gray), alternative conformation (black), common to both conformations (dark gray), interchain contacts from homomers (small circles); predicted contacts (teal) corresponding to experimentally determined structures (opaque circles), predicted contacts not corresponding to experimentally determined structures (noise, translucent diamonds). Panels, top-to-bottom, left-to-right show the experimentally determined contacts underlaying: GREMLIN predictions from the original MSA (Original GMN) and the shallowest subfamily MSA (GMN subfamily, 21 indicates minimum %sequence identity to query); MSA Transformer predictions from the original MSA (Original MSATR) and the shallowest subfamily MSA (MSATR subfamily, 24 indicates minimum %sequence identity to query); predictions from our approach after noise filtering (Full Pipeline) and before noise filtering (Superposition). Predicted contacts from each panel are tabulated by overlap with experimentally determined contacts in the bar graph: dominant (light gray); alternative (black); common (dark gray); noise (teal). Predicted contacts from GREMLIN and MSA Transformer are often redundant; signal increases from our approach are shown in Supplementary Figure 12.

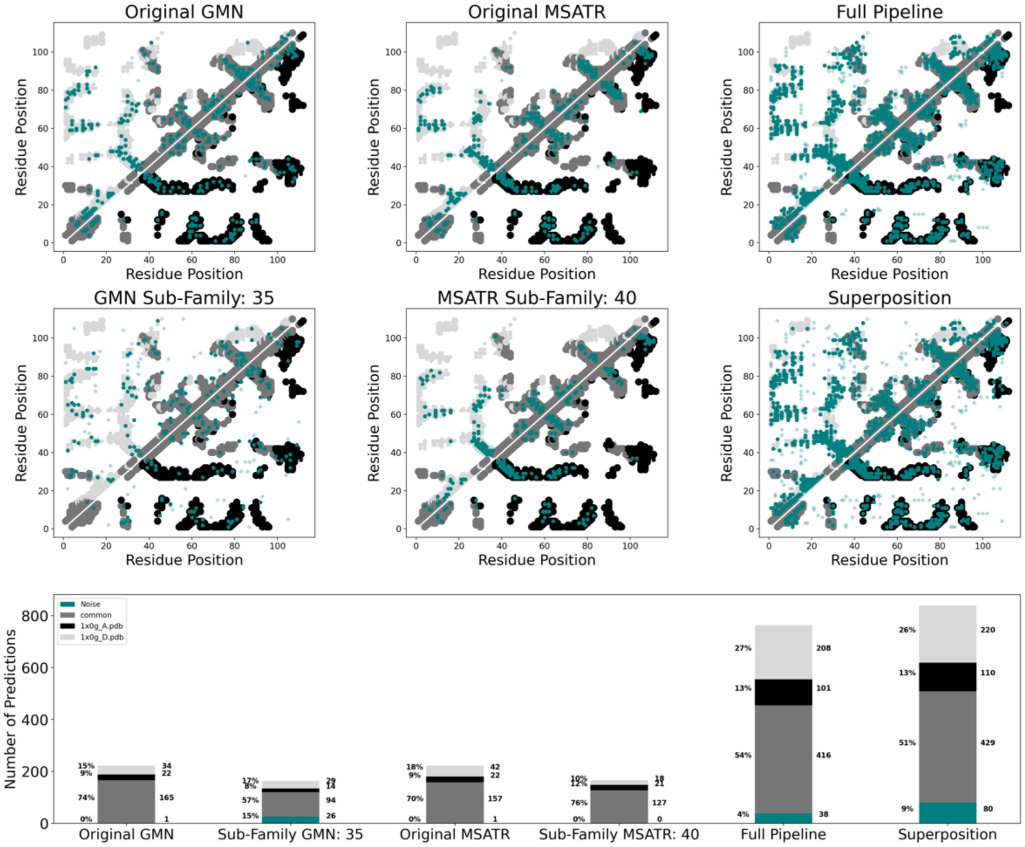

Supplementary Figure 7: Our approach enhances predictions of coevolved amino acid pairs: example 7 of 10. All six upper panels show predicted contacts referenced against contacts from two experimentally determined structures of IscA with PDB codes 1X0G chain D (dominant conformation) and 1X0G chain A (alternative conformation). Color schemes of these six panels follow: experimentally determined contacts unique to the dominant conformation (light gray), alternative conformation (black), common to both conformations (dark gray), interchain contacts from homomers (small circles); predicted contacts (teal) corresponding to experimentally determined structures (opaque circles), predicted contacts not corresponding to experimentally determined structures (noise, translucent diamonds). Panels, top-to-bottom, left-to-right show the experimentally determined contacts underlaying: GREMLIN predictions from the original MSA (Original GMN) and the shallowest subfamily MSA (GMN subfamily, 35 indicates minimum %sequence identity to query); MSA Transformer predictions from the original MSA (Original MSATR) and the shallowest subfamily MSA (MSATR subfamily, 37 indicates minimum %sequence identity to query); predictions from our approach after noise filtering (Full Pipeline) and before noise filtering (Superposition). Predicted contacts from each panel are tabulated by overlap with experimentally determined contacts in the bar graph: dominant (light gray); alternative (black); common (dark gray); noise (teal). Predicted contacts from GREMLIN and MSA Transformer are often redundant; signal increases from our approach are shown in Supplementary Figure 12.

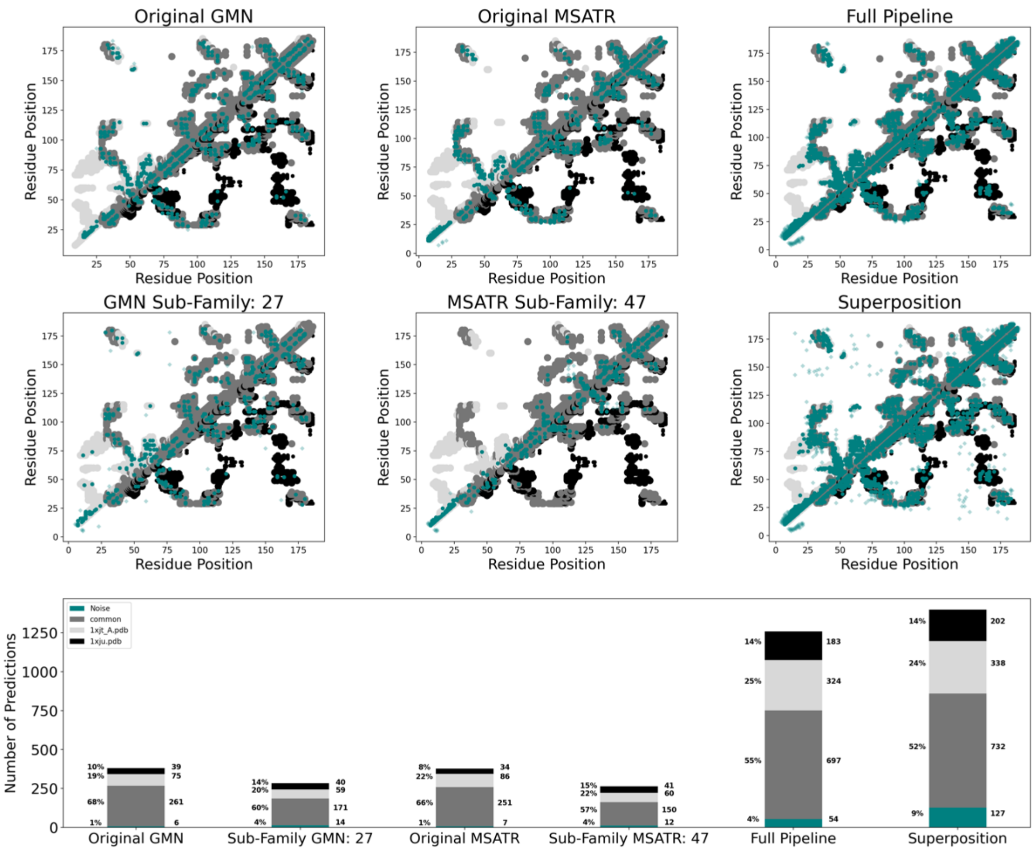

Supplementary Figure 8: Our approach enhances predictions of coevolved amino acid pairs: example 8 of 10. All six upper panels show predicted contacts referenced against contacts from two experimentally determined structures of Endolysin with PDB codes 1XJT (dominant conformation) and 1XJU (alternative conformation). Color schemes of these six panels follow: experimentally determined contacts unique to the dominant conformation (light gray), alternative conformation (black), common to both conformations (dark gray), interchain contacts from homomers (small circles); predicted contacts (teal) corresponding to experimentally determined structures (opaque circles), predicted contacts not corresponding to experimentally determined structures (noise, translucent diamonds). Panels, top-to-bottom, left-to-right show the experimentally determined contacts underlaying: GREMLIN predictions from the original MSA (Original GMN) and the shallowest subfamily MSA (GMN subfamily, 38 indicates minimum %sequence identity to query); MSA Transformer predictions from the original MSA (Original MSATR) and the shallowest subfamily MSA (MSATR subfamily, 44 indicates minimum %sequence identity to query); predictions from our approach after noise filtering (Full Pipeline) and before noise filtering (Superposition). Predicted contacts from each panel are tabulated by overlap with experimentally determined contacts in the bar graph: dominant (light gray); alternative (black); common (dark gray); noise (teal). Predicted contacts from GREMLIN and MSA Transformer are often redundant; signal increases from our approach are shown in Supplementary Figure 12.

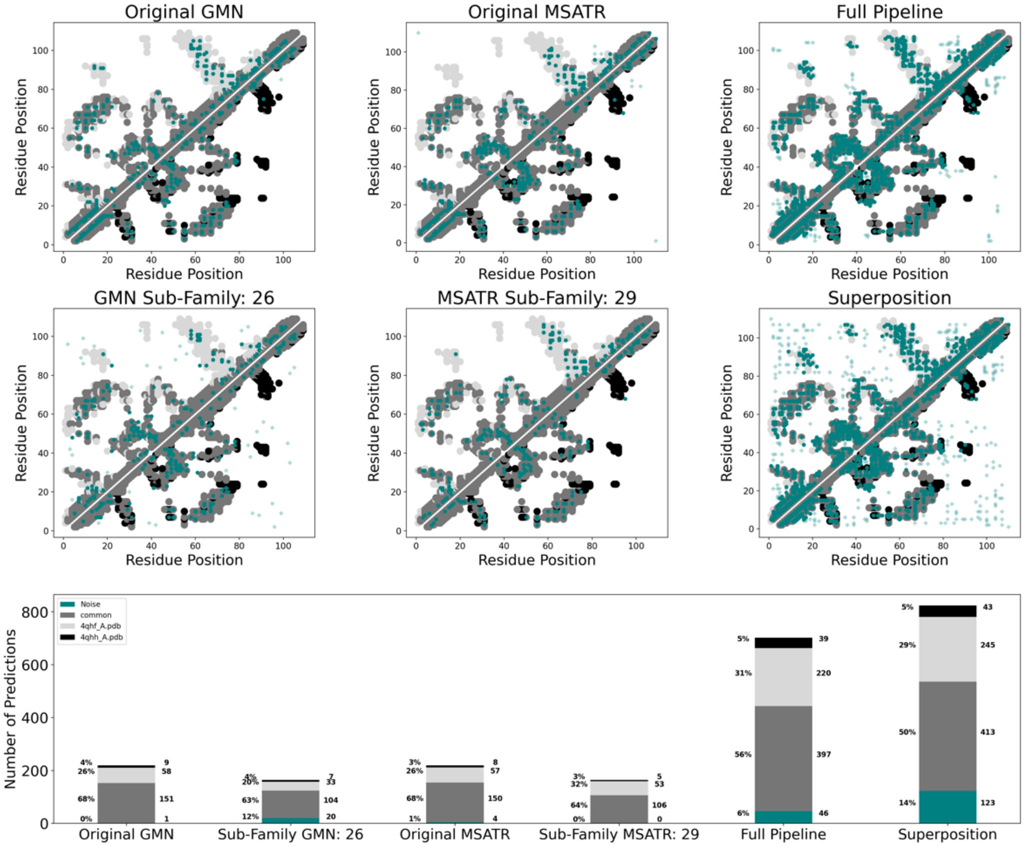

Supplementary Figure 9: Our approach enhances predictions of coevolved amino acid pairs: example 9 of 10. All six upper panels show predicted contacts referenced against contacts from two experimentally determined structures of Selecase with PDB codes 4QHF (dominant conformation) and 4QHH (alternative conformation). Color schemes of these six panels follow: experimentally determined contacts unique to the dominant conformation (light gray), alternative conformation (black), common to both conformations (dark gray), interchain contacts from homomers (small circles); predicted contacts (teal) corresponding to experimentally determined structures (opaque circles), predicted contacts not corresponding to experimentally determined structures (noise, translucent diamonds). Panels, top-to-bottom, left-to-right show the experimentally determined contacts underlaying: GREMLIN predictions from the original MSA (Original GMN) and the shallowest subfamily MSA (GMN subfamily, 25 indicates minimum %sequence identity to query); MSA Transformer predictions from the original MSA (Original MSATR) and the shallowest subfamily MSA (MSATR subfamily, 28 indicates minimum %sequence identity to query); predictions from our approach after noise filtering (Full Pipeline) and before noise filtering (Superposition). Predicted contacts from each panel are tabulated by overlap with experimentally determined contacts in the bar graph: dominant (light gray); alternative (black); common (dark gray); noise (teal). Predicted contacts from GREMLIN and MSA Transformer are often redundant; signal increases from our approach are shown in Supplementary Figure 12.

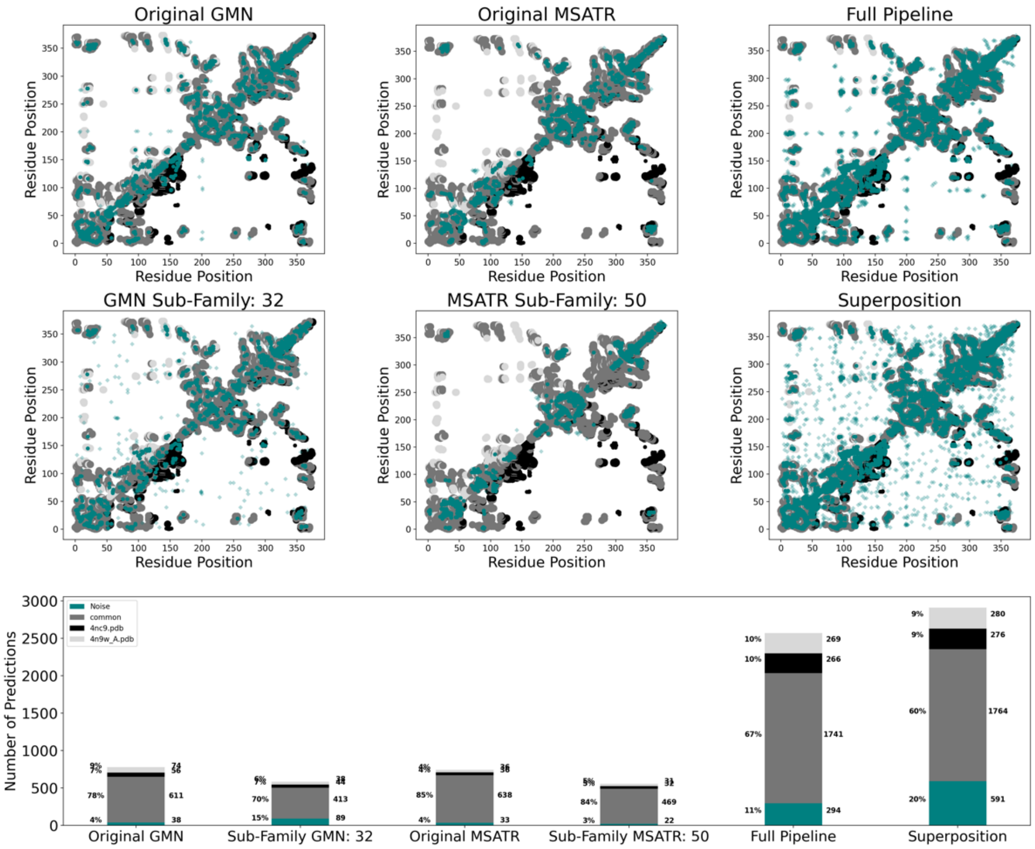

Supplementary Figure 10: Our approach enhances predictions of coevolved amino acid pairs: example 10 of 10. All six upper panels show predicted contacts referenced against contacts from two experimentally determined structures of PimA with PDB codes 4N9W (dominant conformation) and 4NC9 (alternative conformation). Color schemes of these six panels follow: experimentally determined contacts unique to the dominant conformation (light gray), alternative conformation (black), common to both conformations (dark gray), interchain contacts from homomers (small circles); predicted contacts (teal) corresponding to experimentally determined structures (opaque circles), predicted contacts not corresponding to experimentally determined structures (noise, translucent diamonds). Panels, top-to-bottom, left-to-right show the experimentally determined contacts underlaying: GREMLIN predictions from the original MSA (Original GMN) and the shallowest subfamily MSA (GMN subfamily, 31 indicates minimum %sequence identity to query); MSA Transformer predictions from the original MSA (Original MSATR) and the shallowest subfamily MSA (MSATR subfamily, 49 indicates minimum %sequence identity to query); predictions from our approach after noise filtering (Full Pipeline)

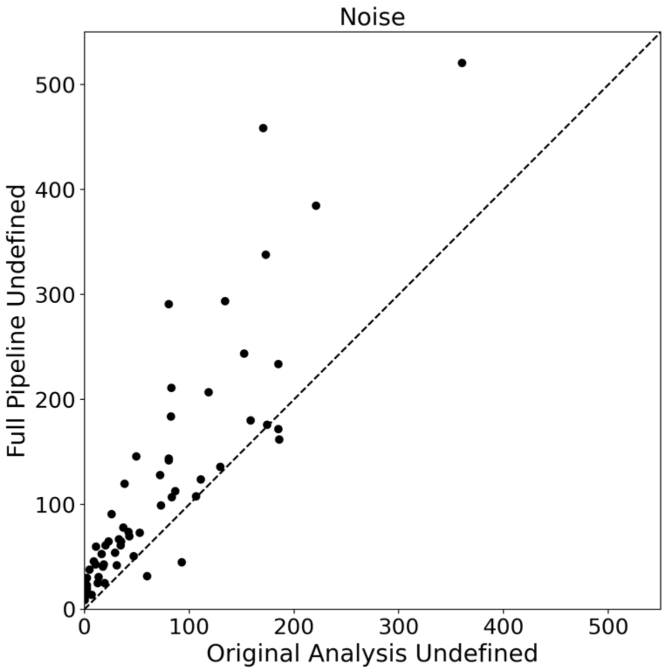

Supplementary Figure 11 Comparison of noise from our pipeline with noise from coevolutionary analysis (GREMLIN+MSA Transformer) on deep MSAs only. Mean/median noise increases were 47%/42%, significantly less than the increase in alternative contacts (mean/median 200%/187%, Supplementary Figure 12a).

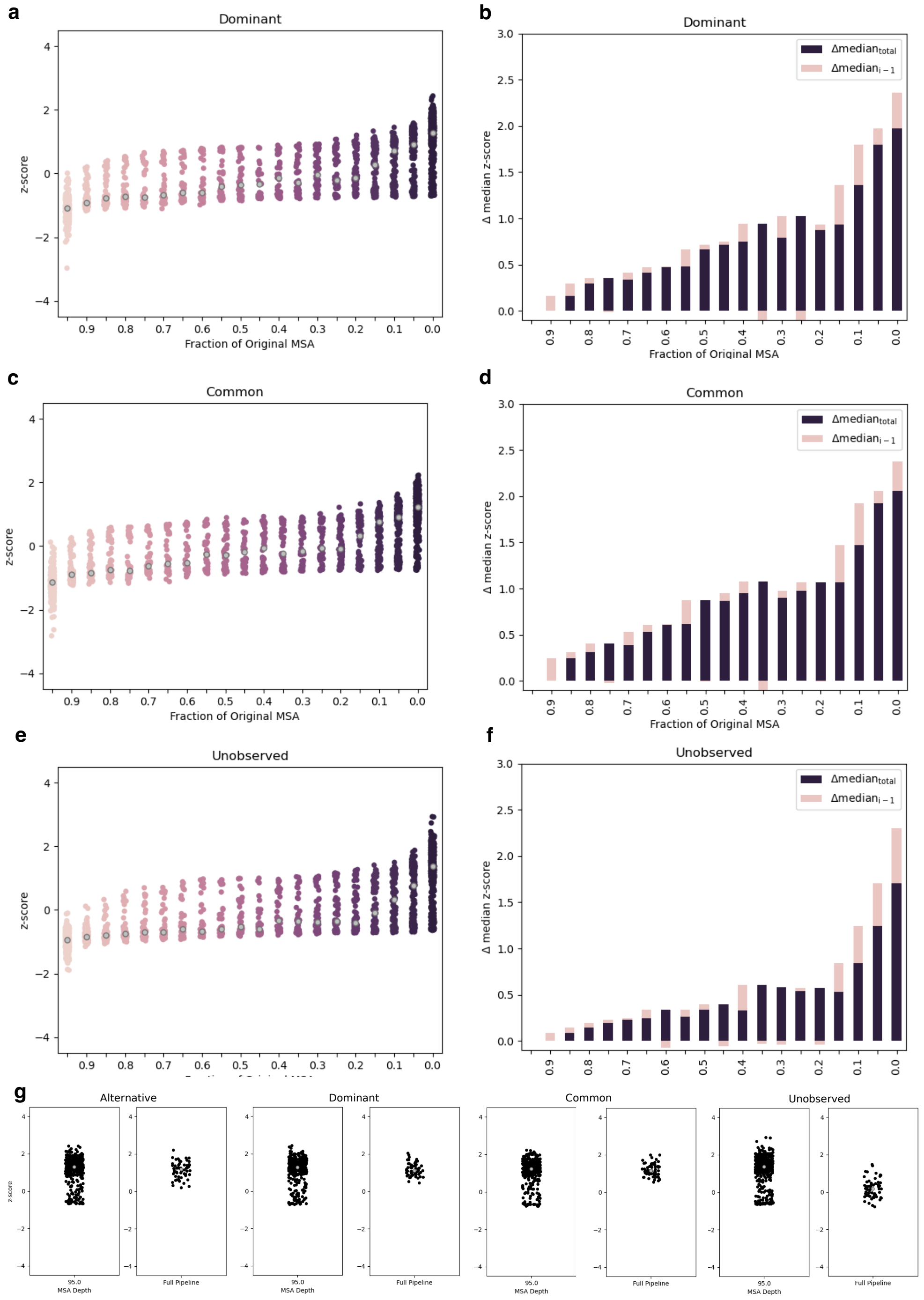

Supplementary Figure 12. Contact predictions are enhanced by subfamily MSAs. Distributions of z-scores of predicted contacts binned by MSA-depth for 56 fold-switching proteins are categorized by Dominant fold (fold with more unique contacts predicted by conventional coevolution, ***a****), Common fold (regions of the protein with the same conformation shared by both folds,* ***c****), and Unobserved (predicted contacts that have not been observed experimentally,* ***e****). Median z-scores of each bin are gray. Bar graphs showing changes in z-scores as a function of bin size are shown for Dominant (****b****), Common (****d****), and Unobserved (****f****) predicted contacts. Purple bars are differences between median z-score of bin (gray dots in (****a,c,e****)) and median z-score of the deepest MSA. Pink bars are differences between median z-score of bin and median z-score of next deepest bin. (****g****). Filtering noise substantially impacts the z-scores of unobserved contacts but has little impact on the z-scores of Alternative, Dominant, and Common contacts.*

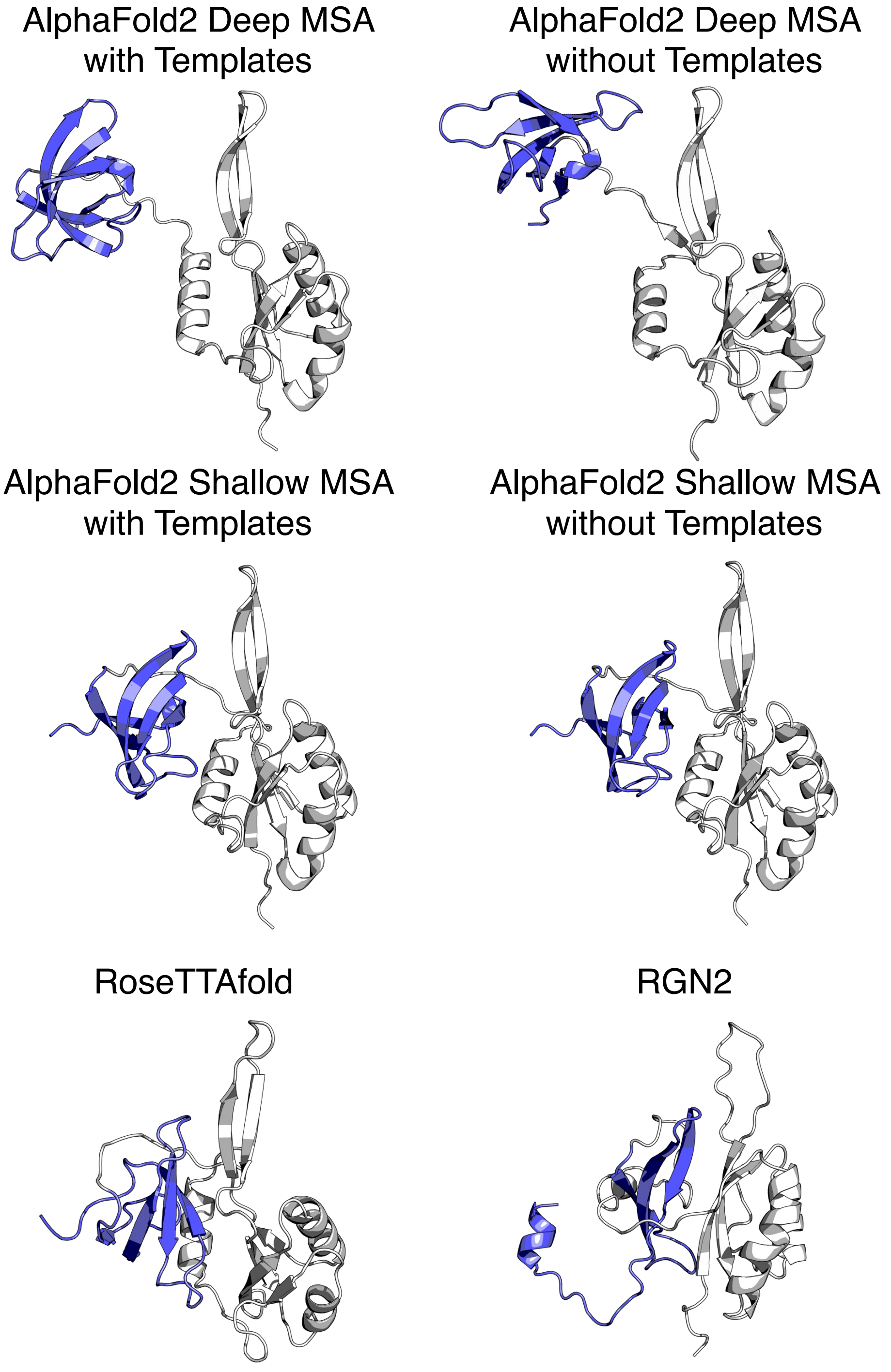

Supplementary Figure 13. State-of-the-art methods predict that the C-terminal domain of Variant 5 (blue) assumes conformations mostly composed of β-sheets. Highest ranked conformers are shown, though the top 5 models from each run all show similar β-sheet structures. The single-folding N-terminal domain of Variant 5 is shown in white.

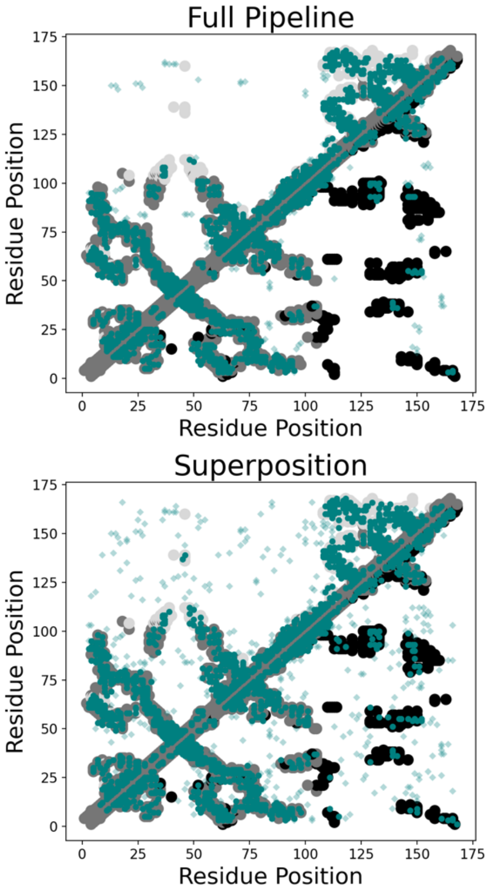

Supplementary Figure 14. Dual-fold coevolution predictions overlap with both AlphaFold2 models of Variant 5. Predicted contacts (teal) unique to the β-roll fold are shown on the upper diagonal (overlapping with light gray contacts from predicted struture), while contacts unique to the α-helical fold are shown on the lower diagonal (overlapping with black contacts from other predicted structures). Contacts predicted in both AlphaFold2 structures are gray. Overlap can be seen after running the full pipeline with noise reduction (4% noise); more overlap, especially with the α-helical fold, can be seen prior to noise reduction (12% noise).

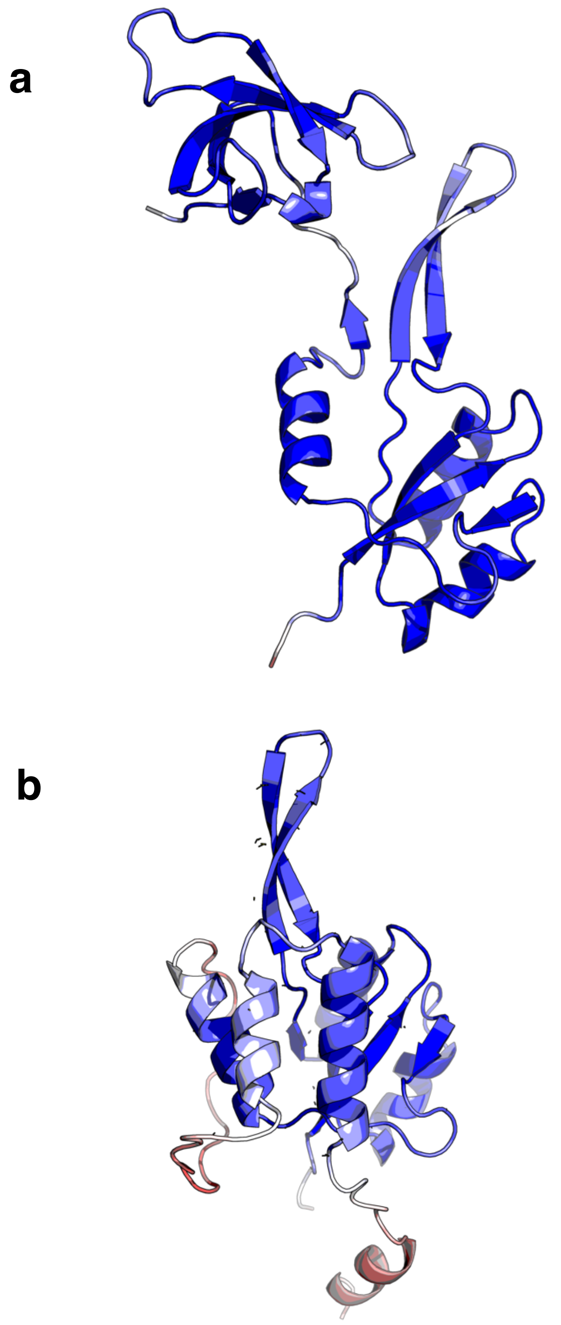

Supplementary Figure 15. AlphaFold2 prediction confidences (pLDDT scores) for the β-roll (a) and α-helical (b) conformations of Variant 5. Most confident scores are dark blue; least confident, red, moderate scores are white.

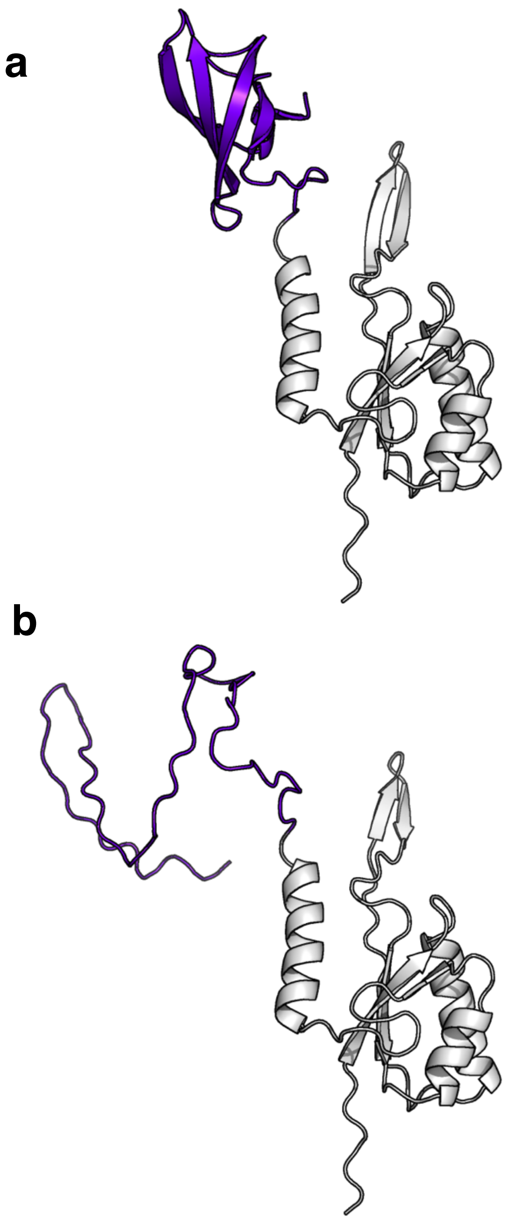

Supplementary Figure 16. Eliminating unique β-sheet coevolutionary signals causes AlphaFold2 to predict an unfolded C-terminal domain (CTD) in single-folding E. coli NusG. AlphaFold2 correctly predicts the ground-state β-roll fold of NusG's CTD (purple) when supplied with an unmodified multiple sequence alignment (MSA, a). Masking coevolutionary signals unique to the β-roll fold causes AlphaFold2 to predict that NusG's CTD is unfolded (b). Folded N-terminal domain is colored gray in both figures.

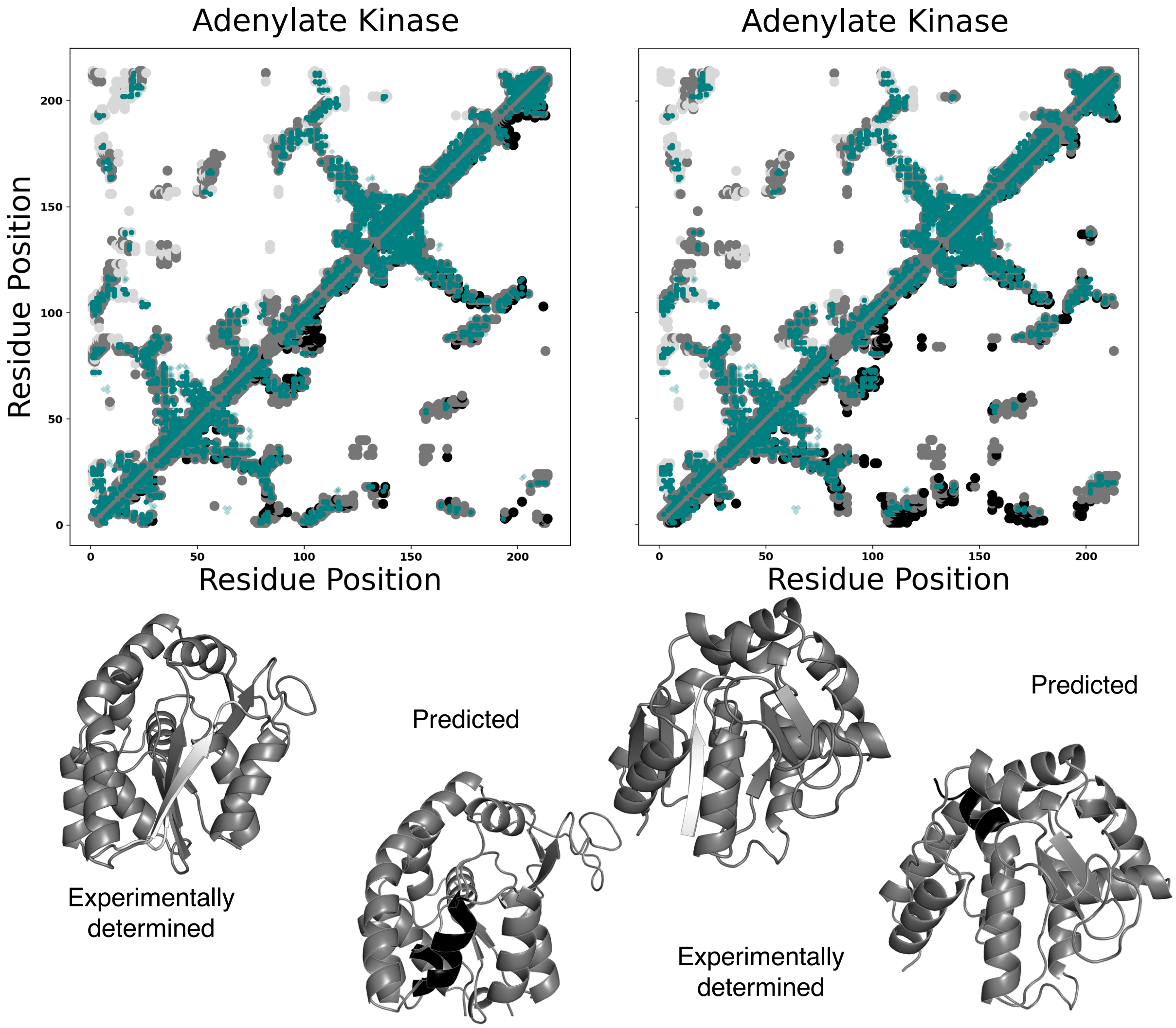

Supplementary Figure 17. Some AlphaFold2-based fold-switch predictions based on modified multiple sequence alignments (MSAs) lack strong coevolutionary signatures. Contact maps of two fold-switching predictions in Adenylate Kinase show the experimentally determined structure on the top diagonal and the ColabFold-predicted fold switched structure on the bottom. ColabFold is an efficient implementation of AlphaFold2 that generates comparable structure predictions. Many predicted coevolved contacts (teal) overlap with contacts unique to the experimentally determined structures (light gray), but few overlap with contacts unique to the alternative structures predicted by ColabFold (black). Structures of both sets of conformations are shown below their respective contact maps. Medium gray regions are common to both folds; white/black correspond to experimentally determined/AF2 prediction. PDB ID of the experimentally determined structure is 4AKE, chain A, shown in different orientations to highlight putative fold-switching regions.

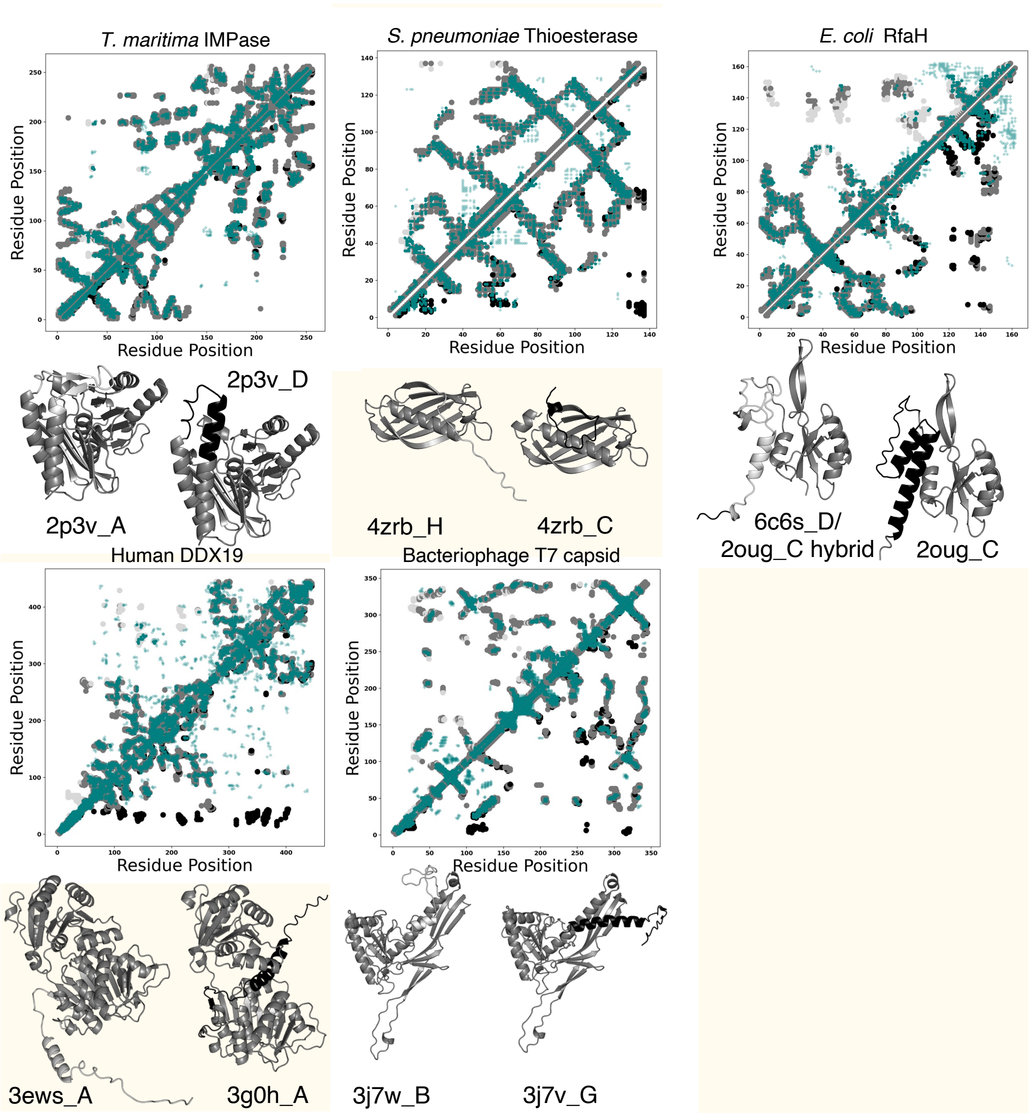

Supplementary Figure 18. Blind predictions of fold-switching proteins. Blind predictions are performed by using ColabFold and ESM-fold to each predict a structure of an amino acid sequence. ACE predicts coevolved residue pairs using the two predicted structures as references. The predicted structures are compared. Different structure predictions both consistent with coevolutionary predictions fall into Category 1, above. Predicted structures are labeled by the PDB IDs and chains to which the correspond most closely. contact maps are shown above structures predicted by ColabFold (fold-switching regions light gray) and ESM-Fold (fold-switching regions black). Predicted contacts are teal.

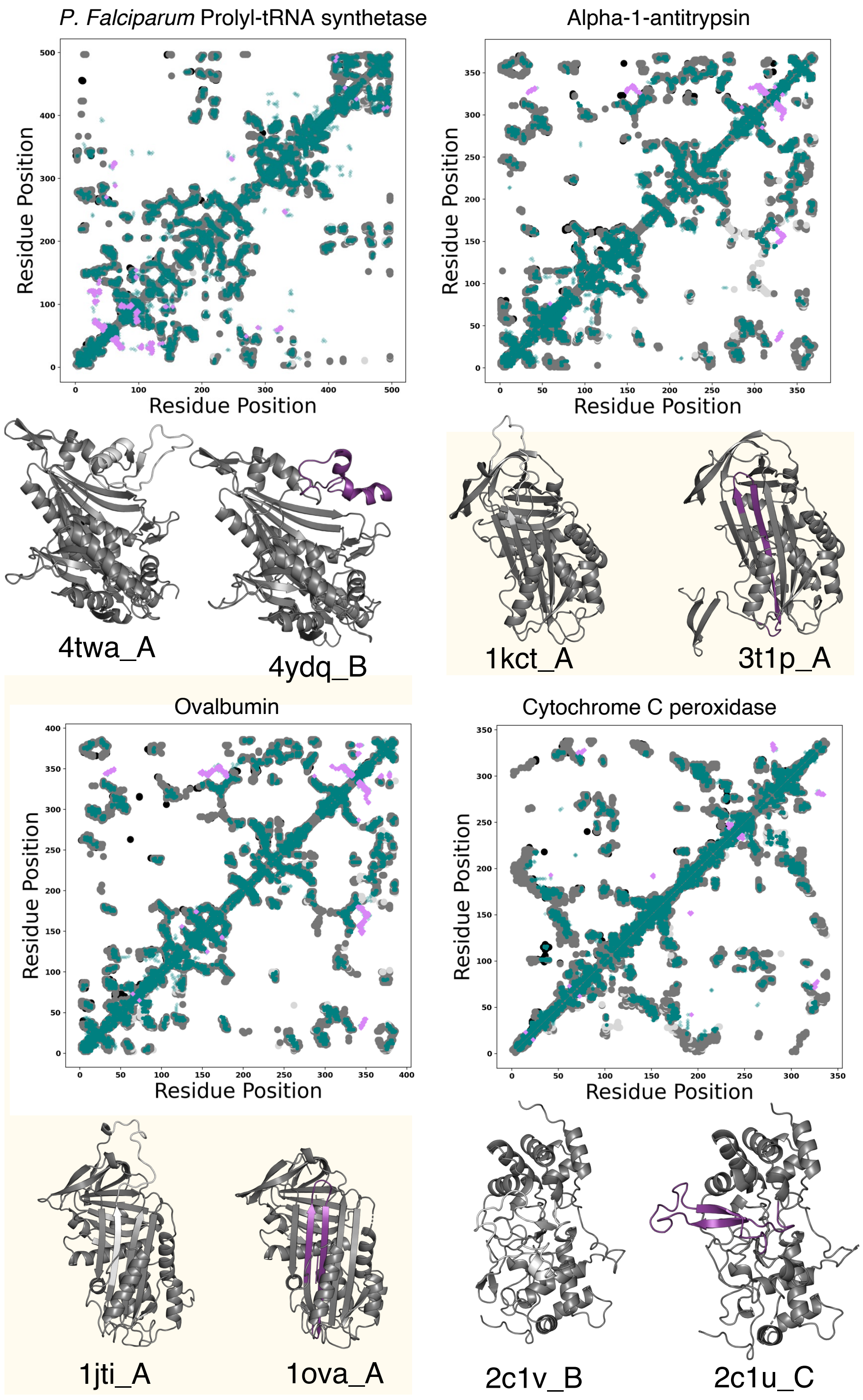

Supplementary Figure 19. Blind predictions of fold-switching proteins. Blind predictions are performed by using ColabFold and ESM-fold to each predict a structure of an amino acid sequence. ACE predicts coevolved residue pairs using the two predicted structures as references. The predicted structures are compared. Similar structure predictions with coevolutionary evidence for an alternative conformation fall into Category 2, above. Predicted structures are labeled by the PDB IDs and chains to which the correspond most closely. Contact maps are shown above structures predicted by ColabFold (fold-switching regions light gray) and ESM-Fold (fold-switching regions black). Predicted contacts are teal. ColabFold and ESM-Fold predict the same conformation. Predicted contacts corresponding to the experimentally characterized alternative conformation are orchid. Structurally conserved protein regions/common contacts are medium gray.

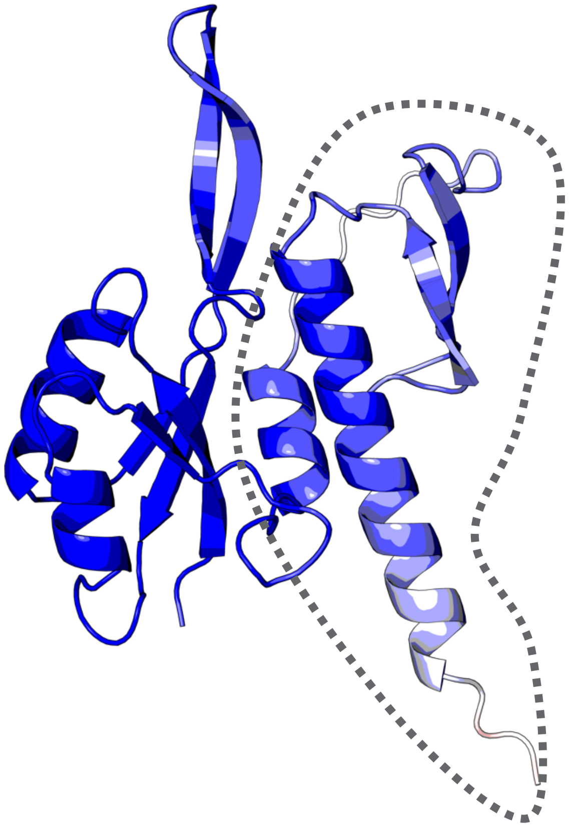

Supplementary Figure 20. High-confidence AlphaFold2 predictions from modified MSAs can be unreliable. Running AlphaFold2 on the shallowest E. coli RfaH MSA used in our coevolutionary analysis yielded in incorrect prediction with high confidence (ranked 0): a CTD with mixed α-helix and β-sheet character (surrounded by gray dots). Structure is colored by prediction confidence: most confident scores are dark blue; least confident, red, moderate scores are white.

*Supplementary Table 1: Structural information and pipeline output for 56 fold-switching proteins. PDBs A and B (PDB ID_Chain) were used to calculate experimentally determined contacts for a given protein; sequences of blue codes were used as queries for generating deep MSAs. Name is the common name of each protein. MSA Depth is the number of sequences in the deepest MSA prepared for GREMLIN and MSA Transformer, and Neff is the number of effective sequences in the deepest MSA. L is the number of amino acids in the query sequence. GMN_QID and MSATR_QID are the number of subfamily alignments made from the original MSA deep enough to run each algorithm. P-values are calculated using the one-tailed hypergeometric test and represent the significance of the additional structural information obtained from the subfamily alignments. One p-value with asterisk was taken for 5*L contacts, due its very short length, rather than 7.5*L as for the rest (Methods).*

PDB A	PDB B	Name	MSA Depth	Neff	L	GMN_QID	MSATR_QID	P-value
1k0n_A	1rk4_A	CLIC1	53571	30669.7	198	14	17	0
2p3v_A	2p3v_D	IMPase	51161	32813.9	248	26	28	0
5jyt_A	4kso_A	KaiB	40770	24766.0	90	22	41	0
4zrb_C	4zrb_H	Thioesterase	32221	18274.6	126	21	22	2.18E-174
1jfk_A	2nxq	CaBP	28719	17789.4	131	21	25	3.34E-217
2k0q_A	2lel_A	CopK	28288	24441.6	67	18	22	5.08E-30
1miq_B	1qs8_B	Proplasmepsin	23159	15978.4	320	20	27	0
1nqd_A	1nqj_B	CBD	22522	19969.0	113	21	24	4.14E-199
1x0g_A	1x0g_D	IscA	21882	10482.2	106	31	33	1.28E-128
4gqc_B	4gqc_C	PrxQ	20175	10909.3	151	23	26	3.85E-298
2c1u_C	2c1v_B	Cytochrome C Peroxidase	19035	12824.6	308	30	36	0
2uy7_D	5flu_E	papA	18658	13019.7	152	23	28	1.85E-284
2jmr_A	4j3o_F	fimF	18642	13231.8	150	25	28	5.09E-244
3low_A	3m1b_F	Beta-2-Microglobulin	17286	9085.7	96	29	38	1.40E-77
5ond_A	6c6s_D	RfaH	17278	10318.5	160	25	32	1.01E-232
4pyi_A	4pyj_A	COMT	16360	10138.6	208	22	33	6.52E-288
4qhf_A	4qhh_A	Selecase	15077	9500.2	101	17	21	2.39E-89
1jti_A	1ova_A	Ovalbumin	15075	10168.2	372	23	32	0
1mnm_C	1mnm_D	MCM1	14095	5811.2	72	23	26	3.42E-48
3uyi_A	3v0t_A	Perakine Reductase	13385	6113.7	324	17	19	0
5f3k_A	5f5r_B	hTrap1	11089	5132.3	212	30	34	6.78E-224
2mwf_A	2nnt_A	amyloid protofilament	11073	6125.0	36	44	46	0.0091*
1uxm_K	2nam_A	SOD1	10984	6634.0	149	36	36	0
4aal_A	4aan_A	MacA	10597	6940.6	312	21	26	0
3j7v_G	3j7w_B	Capsid Protein	8922	6322.3	308	11	13	0
3ejh_A	3m7p_A	FN1	8708	4536.3	87	23	31	4.28E-293
3hde_A	3hdf_A	Lysozyme	8612	5882.3	149	29	36	3.88E-303
2kxo_A	3r9j_C	MinE	8461	3963.8	83	42	48	6.02E-63
2axz_A	2grm_B	PrgX	8259	6699.6	301	11	15	0
1kct_A	3t1p_A	Alpha-1-Antitrypsin	8073	4982.4	368	14	22	0

2qqj_A	4qds_A	NRP2	8058	5002.7	166	22	25	0
4twa_A	4ydq_B	PRS	7206	3394.2	477	15	18	0
4xws_D	4y0m_J	OxyR	7202	4156.6	218	18	28	1.04E-171
3ifa_A	5et5_A	FBP2	6958	3372.0	328	24	30	0
1rep_C	2z9o_B	RepE	6279	4580.3	239	17	21	0
2wcd_X	4phq_A	ClyA	5924	4387.3	289	8	16	0
4n9w_A	4nc9_C	PimA	5794	3989.8	371	17	35	0
4o01_D	4o0p_A	bphP	5427	3552.0	495	12	14	0
1xjt_A	1xju_B	lysa	4800	3397.4	156	18	27	1.28E-298
5c1v_A	5c1v_B	CALNA	4206	2071.1	301	13	17	1.18E-308
1rkp_A	2h44_A	PDE5A1	4117	2732.1	315	16	29	0
2lep_A	4hdd_A	GlpG	4114	3322.2	74	28	37	2.61E-23
5b3z_A	5bmy_A	hPin1	3871	2402.1	389	8	10	0
2hdm_A	2n54_B	XCL1	3137	1968.2	68	24	34	1.68E-52
4rwn_A	4rwq_B	OAS1	2866	1880.4	342	7	26	0
4q79_F	4uv2_D	CsgG	2635	1526.4	250	11	33	0
4dxr_A	4dxt_A	SUN2	2525	1711.3	192	23	36	1.52E-138
2vfx_L	3gmh_L	Mad2	2501	1622.1	201	12	36	8.36E-131
1cee_B	2k42_A	WASP	1679	1027.3	56	26	40	3.41E-49
3zwg_N	4tsy_D	FraC	1198	863.4	170	7	21	6.08E-237
1zk9_A	3jv6_A	RelB	1121	578.9	99	16	29	0
4jph_B	5hk5_H	Grem2	978	426.8	120	6	17	4.68E-124
1ebo_E	5fhc_J	viral fusion protein	869	496.8	92	8	21	5.57E-62
1iyt_A	2nao_F	beta-amyloid protein	780	206.6	33	15	19	2.70E-08
3ews_A	3g0h_A	DDX19	5484	2478.6	390	9	23	0
2ged_A	1nrj_B	SRP	4022	2036.0	165	15	20	4.19E-139

*Supplementary Table 2. Correspondence between experimentally observed and dominant contacts. Conformations are ordered to be experimentally consistent; protein pairs whose predictions do not match experiment are highlighted orange. Conformations equally populated at equilibrium are marked with **. PDB ID format: ID_Chain.*

Protein Pair	Dominant	Alternative	Protein Pair	Dominant	Alternative
1	2k42_A	1cee_B	30	3ejh_A	3m7p_A
2	5fhc_J	1ebo_E	31	3hde_A	3hdf_A
3	1iyt_A	2nao_F	32	5et5_A	3ifa_A
4	1jfk_A	2nxq_B	33	3j7w_B**	3j7v_G**
5	1jti_A	1ova_A	34	3m1b_F	3low_A
6	1k0n_A	1rk4_B	35	3uyi_A	3v0t_A
7	1kct_A	3t1p_A	36	3zwg_N	4tsy_D
8	1qs8_B	1miq_B	37	4aan_A	4aal_A
9	1mnm_D**	1mnm_C**	38	4dxt_A	4dxr_A
10	1nqj_B	1nqd_A	39	4gqc_C	4gqc_B
11	1rep_C	2z9o_B	40	4jph_B	5hk5_H
12	2h44_A	1rkp_A	41	4n9w_A	4nc9_C
13	4kso_A	5jyt_A	42	4o0p_A	4o01_D
14	1uxm_K	2nam_A	43	4pyi_A	4pyj_A
15	1x0g_D**	1x0g_A**	44	4uv2_D	4q79_F
16	1xjt_A	1xju_B	45	4qhf_A	4qhh_A
17	3jv6_A	1zk9_A	46	4rwn_A	4rwq_B
18	2grm_B	2axz_A	47	4twa_A	4ydq_B
19	2hdm_A**	2n54_B**	48	4y0m_J	4xws_D
20	2jmr_A	4j3o_F	49	4zrb_C	4zrb_H
21	2k0q_A	2lel_A	50	5b3z_A	5bmy_A
22	2kxo_A	3rj9_C	51	5c1v_A	5c1v_B
23	2lep_A	4hdd_A	52	5f5r_B	5f3k_A
24	2mwf_A	2nnt_A	53	5ond_A	6c6s_D
25	2p3v_A**	2p3v_D**	54	2c1v_B	2c1u_C
26	2qqj_A	4qds_A	55	3ews_A	3g0h_A
27	2uy7_D	5flu_E	56	2ged_A	1nrj_B
28	2vfx_L**	3gmh_L**			
29	4phq_A	2wcd_X			
